# Phenotype detection of metallo-β-lactamase among the imipenem resistant *Psudomonas* and *Acinetobacter* in the tertiary care hospitals of Dhaka city

**Published:** 2011-01-10

**Authors:** Shaheda Anwar, Ruhul Amin

**Affiliations:** 1 Department of Microbiology, Bangabandhu Sheikh Mujib Medical University, Dhaka-1000, Bangladesh

## 

Metallo beta lactamases (MBL) are enzymes that have wide spread of activity and they confer a high level of resistance to all β-lactams including carbapenem, except aztreonam [1]. MBLs require divalent zinc ion for their enzymatic activity which is not diminished by serine β lactamase inhibitors like salbactam, tazobactam, clavulinic acid etc but is inhibited by metal chelators like EDTA and thiol based compounds such as 2-mercaptopropionic acid (2-MPA), 10-phenanthroline, calcium dipicholinate etc [2]. They have constant and efficient carbapenemase activity. This MBL production is typically associated with resistance to aminoglycosides and fluoroquinolones further compromising therapeutic options [3].

There are no standard methods for the detection of MBL production in gram negative organism in routine Microbiology practice. The present study was undertaken to evaluate the screening tests like double disk synergy test (DDST) and disk potentiation test (DPT) using ceftazidime (CAZ) and imipenem (IPM) disks with chelating agents like EDTA and 2-MPA. A total of 132 *Pseudomonas* and 76 *Acinetobacter* isolates were obtained from Bangabandhu Sheikh Mujib Medical University (BSMMU), Bangladesh Institute of Research and Rehabilitation for Diabetes, and Endocrine and Metabolic disorders (BIRDEM) hospitals of Dhaka city. A total of 53 and 29 IPM resistant *Pseudomonas* and *Acinetobacter* isolates respectively were selected. EDTA- IPM micro dilution minimum inhibitory concentration (EDTA-IPM micro dilution MIC) method detected MBL in 44 (83%) IPM resistant *Pseudomonas* and 19(65.5%) *Acinetobacter* isolates. DDST with CAZ-0.1M EDTA and CAZ-2-MPA detected MBL in 73.6% and 67.9% of IPM resistant *Pseudomonas* and 55.2% and 48.3% of *Acinetobacter* isolates respectively. The detection rate was 67.9% and 66.1% in *Pseudomonas* and 51.7% and 44.8% in *Acinetobacter* isolates by EDTA-IPM and IPM-2-MPA methods respectively. In comparison to DDST, DPT with 0.1M EDTA showed higher sensitivity (89.7%) and specificity (100%) for detection of MBL in *Pseudomonas* and *Acinetobacter.* Isolates were also tested for AmpC β lactamase by DPT using chelating agent - aminophenyl boronic acid (APB) and it detected AmpC β lactamase in 6.9% and 21.1% MBL positive *Pseudomonas* and *Acinetobacter* respectively.

MBL producing *Pseudomonas* and *Acinetobacter* are emerging in our country. Rapid detection of these MBLs is necessary to institute appropriate treatment and effective infection control measures. Simple screening test like DPT using CAZ/IPM with 0.1M EDTA can be introduced into the routine clinical laboratories for their early detection.

## References

[B1] Noyal M Menezes G Harish B Sujatha S Panja S Simple screening tests for detection of carbapenemases in clinical isolates of nonfermentative Gram negative bacteria Indian J Med Res 2009 129 707 712 19692754

[B2] Livermore DM Woodford N Carbapenemases: a problem in waiting? Curr Opin Microbiol 2000 3 489 495 10.1016/S1369-5274(00)00128-4 11050448

[B3] Walsh TR The emergence and implications of metallo-beta-lactamases in Gram negative bacteria Clin Microbiol Infect 2005 11 2 9 10.1111/j.1469-0691.2005.01264.x 16209700

